# Clinical contribution of mNGS in the diagnosis and management of imported falciparum malaria in a malaria-free region: a five-case series from northern China

**DOI:** 10.3389/fcimb.2026.1978670

**Published:** 2026-09-24

**Authors:** Bin Niu, Rong Wang, Haiyan Tian, Xinran Li, Yangdi Yu, Jiaolong Ren, Liaoyun Zhang

**Affiliations:** 1 Department of Infectious Diseases, The First Hospital of Shanxi Medical University, Taiyuan, China; 2 The First Clinical Medical School, Shanxi Medical University, Taiyuan, China

**Keywords:** clinical management, imported malaria, malaria-free region, metagenomic next-generation sequencing, *Plasmodium falciparum*

## Abstract

**Background:**

Although malaria transmission has been eliminated in China, imported *Plasmodium falciparum* malaria remains a diagnostic challenge, particularly in non-endemic regions where clinical experience is limited. The nonspecific manifestations and potential for rapid progression to severe disease may result in delayed diagnosis and inappropriate management. Metagenomic next-generation sequencing (mNGS) has emerged as a complementary diagnostic approach for complex infectious diseases; however, its role in imported malaria management remains insufficiently characterized.

**Methods:**

We retrospectively reviewed five patients with imported *P. falciparum* malaria in whom mNGS contributed to diagnosis or clinical management in a malaria-free region of northern China. Demographic characteristics, epidemiological exposure, clinical manifestations, laboratory findings, mNGS results, treatment strategies, and outcomes were analyzed. Clinical scenarios in which mNGS provided additional diagnostic information were summarized.

**Results:**

All patients had travel or residence histories in malaria-endemic regions in Africa. mNGS detected *P. falciparum* sequences in all patients, while peripheral blood smear examination demonstrated malaria parasites, with sequencing reads ranging from 121 to 212,513. Clinical presentations ranged from uncomplicated febrile illness to severe malaria with multiorgan involvement, including impaired consciousness, severe anemia, thrombocytopenia, and acute kidney injury requiring continuous renal replacement therapy. In four patients, mNGS was incorporated into early etiological evaluation, whereas in one patient it was used for reassessment of recurrent fever and suspected concomitant infection. In this patient, mNGS provided additional evidence supporting malaria-related evaluation without identifying clinically significant alternative pathogens.

**Conclusion:**

Imported *P. falciparum* malaria remains challenging to diagnose and manage in malaria-free regions. This case series highlights that mNGS may provide complementary information beyond pathogen detection, particularly in patients with complex presentations or uncertain clinical trajectories. Integration of mNGS with conventional parasitological methods and clinical assessment may facilitate the management of imported malaria in non-endemic settings.

## 1 Introduction

Although China was certified malaria-free by the World Health Organization in 2021, imported malaria remains an ongoing challenge for sustaining malaria elimination achievements. Recent epidemiological studies have shown that malaria cases reported in China are now predominantly imported infections, with *Plasmodium falciparum* representing a major proportion of cases acquired in Africa (Liu et al., 2024; Zhu et al., 2024). Similar diagnostic challenges persist in other malaria-free regions, where imported malaria continues to occur despite interruption of local transmission (Weiland, 2023).

The diagnosis of imported *P. falciparum* malaria can be challenging in non-endemic settings because clinicians and laboratories have limited exposure to malaria cases. Reduced clinical familiarity may lower diagnostic suspicion, particularly when travel history is overlooked or presentations are nonspecific. Delayed recognition and treatment have been associated with increased risks of severe complications, emphasizing the need to maintain diagnostic awareness after malaria elimination (Bastaki et al., 2018; Weiland, 2023). However, unlike endemic regions where malaria diagnosis and monitoring are routinely performed, non-endemic areas may face additional challenges related to maintaining expertise in malaria recognition and laboratory interpretation.

Peripheral blood smear microscopy remains the cornerstone of malaria diagnosis because it enables direct visualization of parasites, identification of developmental stages, and assessment of parasitemia and parasite clearance during treatment. However, the accuracy and clinical interpretation of microscopy depend on factors including parasite density, sampling timing, and examiner expertise, which may be difficult to maintain in regions where malaria cases are rarely encountered (Calderaro et al., 2021; Li et al., 2021). Although molecular methods such as polymerase chain reaction (PCR) provide high sensitivity, they are not routinely available in many clinical settings and may not provide immediate results for urgent clinical decision-making (Williams and Drennan, 2022). Therefore, imported malaria in non-endemic regions may still present diagnostic and therapeutic challenges despite the availability of established diagnostic approaches.

Metagenomic next-generation sequencing (mNGS) has emerged as a complementary diagnostic approach for clinically challenging infections because it enables broad-range pathogen detection without requiring a predefined pathogen hypothesis. Although previous studies have demonstrated the feasibility of detecting *Plasmodium* species by sequencing, the clinical role of mNGS in imported malaria, particularly its contribution to diagnostic pathways and subsequent management decisions in non-endemic settings, remains insufficiently characterized (Edward and Handel, 2021; Liu and Ma, 2024).

In this study, we retrospectively describe five cases of imported *P. falciparum* malaria diagnosed in a malaria-free region of northern China. By integrating epidemiological information, conventional parasitological examination, mNGS results, treatment courses, and longitudinal clinical changes, we aim to illustrate the potential role of mNGS as a complementary tool for improving pathogen recognition and supporting clinical decision-making in imported malaria occurring in non-endemic settings.

## 2 Materials and methods

### 2.1 Study design and participants

This retrospective case series was conducted at the First Hospital of Shanxi Medical University. Patients diagnosed with imported *P. falciparum* malaria between 2023 and 2026 were retrospectively reviewed. Eligible patients were required to meet the following criteria: (1) epidemiological evidence of exposure to malaria-endemic regions; (2) clinical manifestations compatible with malaria infection; (3) laboratory confirmation of *P. falciparum* infection through integrated evaluation of parasitological examination, molecular testing, and clinical evidence; and (4) availability of complete clinical records.

Patients were excluded if clinical information was incomplete or if malaria infection could not be confirmed by integrated evaluation of clinical presentation, epidemiological history, parasitological examination, and mNGS findings.

This study was conducted in strict accordance with the Declaration of Helsinki, and was approved by the institutional review board of The First Hospital of Shanxi Medical University (Approval No. SYDYY-KYLL-AF-003/01). Informed consent was obtained from all participants. Data supporting the findings of this study are available from the corresponding author upon reasonable request.

### 2.2 Clinical data collection and assessment

The following data were extracted from the electronic medical records: (1) demographic and epidemiological characteristics, including sex, age, occupation, ethnicity, and recent travel or residence in malaria-endemic regions; (2) clinical manifestations, including duration of fever before admission, maximum body temperature, and accompanying symptoms; (3) hematological parameters, including white blood cell count, neutrophil count, red blood cell count, hemoglobin level, and platelet count; (4) inflammatory markers, including interleukin-6 and procalcitonin; (5) biochemical parameters related to hepatic and hemolytic involvement, including alanine aminotransferase, aspartate aminotransferase, alkaline phosphatase, gamma-glutamyl transferase, total bilirubin, direct bilirubin, indirect bilirubin, and lactate dehydrogenase; (6) renal and metabolic parameters, including serum creatinine and glucose; (7) coagulation parameters, including prothrombin time, prothrombin activity, international normalized ratio, activated partial thromboplastin time, D-dimer, and fibrinogen; and (8) treatment and clinical outcomes, including antimalarial regimens and treatment modifications, blood component transfusion, continuous renal replacement therapy (CRRT), length of hospital stay, parasite clearance, recovery of organ dysfunction, and status at discharge.

The severity of malaria was assessed according to the Guidelines for the diagnosis and treatment of malaria in China for severe malaria (National Center for Infectious Diseases Writing Group, 2022). Severe disease was defined by the presence of major organ dysfunction or severe malaria-related complications, including impaired consciousness, severe anemia, thrombocytopenia, AKI, metabolic abnormalities, coagulation disorders, and the need for organ-supportive therapies.

Longitudinal changes in body temperature, hemoglobin concentration, platelet count, and serum creatinine were reviewed during hospitalization to evaluate clinical response and organ recovery.

### 2.3 mNGS sequencing and bioinformatic analysis

Peripheral blood samples were collected according to the clinical diagnostic workflow. After centrifugation, the plasma fraction was separated and used for downstream nucleic acid extraction and mNGS analysis. DNA was extracted using the MGIEasy Genomic DNA Extraction Kit (magnetic bead-based method; Cat. No. 940-000972-00, MGI Tech, China). The extracted DNA was subsequently subjected to library preparation, including enzymatic fragmentation, end repair, adapter ligation, PCR amplification, magnetic-bead purification, and library quality control. Because this assay was based on untargeted metagenomic sequencing, no *Plasmodium falciparum*-specific primers were used; amplification was performed using universal library primers. Qualified libraries were circularized and converted into DNA nanoballs (DNBs), pooled, and sequenced on the MGISEQ-2000 platform (MGI Tech, China) using the MGISEQ-2000RS High-throughput Sequencing Reagent Set (SE50; Cat. No. 1000012551) with a single-end 50-bp sequencing strategy. Positive controls, negative controls, and internal controls were included throughout the sequencing workflow to monitor assay performance and potential contamination.

Raw sequencing data were subjected to quality-control filtering, including removal of low-quality, low-complexity, and adapter-contaminated reads, followed by depletion of human-derived sequences. Bioinformatic analysis was performed using the HALOS PMseq platform (BGI, China). The remaining non-human reads were aligned against the BGI-developed PMDB pathogen reference database, which contains bacterial, fungal, viral, and parasitic reference sequences, for taxonomic identification. Sequencing results were evaluated using multiple parameters, including species-specific mapped reads, relative abundance, genomic coverage, sequencing depth, depth ratio, and Shannon index. The distribution of reads across the reference genome was also reviewed, with broadly and randomly distributed reads considered more supportive of reliable detection. For clinically relevant or unexpected positive findings, representative sequences were independently re-aligned using NCBI BLASTN against the core_nt database to verify taxonomic assignment and reduce the possibility of erroneous classification. Potential pathogens were interpreted after excluding probable background microorganisms, batch-related contamination, and signals potentially attributable to cross-sample interference. Final sequencing reports were jointly reviewed by two experienced mNGS analysts, and any discrepancies in interpretation were resolved through discussion and consensus. The clinical significance of detected microorganisms was subsequently determined by integrating sequencing characteristics with epidemiological exposure, clinical manifestations, peripheral blood smear findings, laboratory abnormalities, and treatment response. A positive mNGS result was not interpreted solely on the basis of sequencing reads but required concordance with species-specific sequence evidence and the overall clinical context.

### 2.4 Conventional parasitological examination

Peripheral blood smear examination was performed as part of the routine diagnostic evaluation for suspected malaria. Thin blood films were prepared from peripheral blood samples and examined microscopically for the presence of malaria parasites. In our clinical setting, microscopy was used primarily for qualitative parasite detection and for documenting visible parasitic forms when present, rather than for definitive species-level identification.

During hospitalization, repeated peripheral blood smear examinations were performed when clinically indicated to assess parasite persistence or clearance. Standardized quantitative parasitemia measurement was not routinely available, and smear examinations were not performed according to a fixed daily schedule. Therefore, microscopy findings were interpreted mainly as qualitative evidence of parasite presence or clearance. Species-level identification of Plasmodium falciparum was based on integration of mNGS findings with epidemiological, clinical, and parasitological evidence rather than on erythrocyte morphology alone.

### 2.5 Treatment and clinical outcome assessment

Antimalarial treatment was administered according to the Guidelines for the diagnosis and treatment of malaria in China (National Center for Infectious Diseases Writing Group, 2022). Because the study was conducted in a non-endemic region where antimalarial drugs were not routinely stocked, the required medications were obtained through coordination with the local Center for Disease Control and Prevention. Intravenous artesunate was used for initial parenteral antimalarial therapy, followed by oral dihydroartemisinin–piperaquine (DHA–PPQ) after clinical stabilization and when oral treatment was considered appropriate. Treatment duration and subsequent modifications were determined according to the clinical course and serial peripheral blood smear findings, including persistence or disappearance of detectable parasites. Additional supportive interventions, including red blood cell and platelet transfusion, CRRT, and other organ-supportive measures, were provided when clinically indicated.

Treatment response and clinical outcomes were assessed using longitudinal clinical and laboratory information. The main outcomes included resolution of fever, clearance of malaria parasites on peripheral blood smear examination, recovery of hematological abnormalities, improvement or recovery of organ dysfunction, length of hospital stay, and survival status at discharge. For patients with severe complications, post-discharge information was reviewed when available to assess subsequent recovery, particularly the course of persistent organ dysfunction.

## 3 Results

### 3.1 Demographic and clinical characteristics

Five patients with imported *P. falciparum* malaria were included in this case series. All patients were male and of Han Chinese ethnicity, with ages ranging from 26 to 51 years. One patient was a farmer, whereas the remaining four were professional or technical workers. All five patients had a recent history of travel to or residence in malaria-endemic regions in Africa, supporting imported rather than locally acquired infection. Specifically, one patient had returned from Côte d’Ivoire, one from Tanzania, and one from Ghana; for the remaining two patients, the country of exposure within Africa could not be further specified. The geographical distribution of the presumed exposure locations and their relationship to Shanxi Province are illustrated in **Figure 1**.

**Figure 1:**
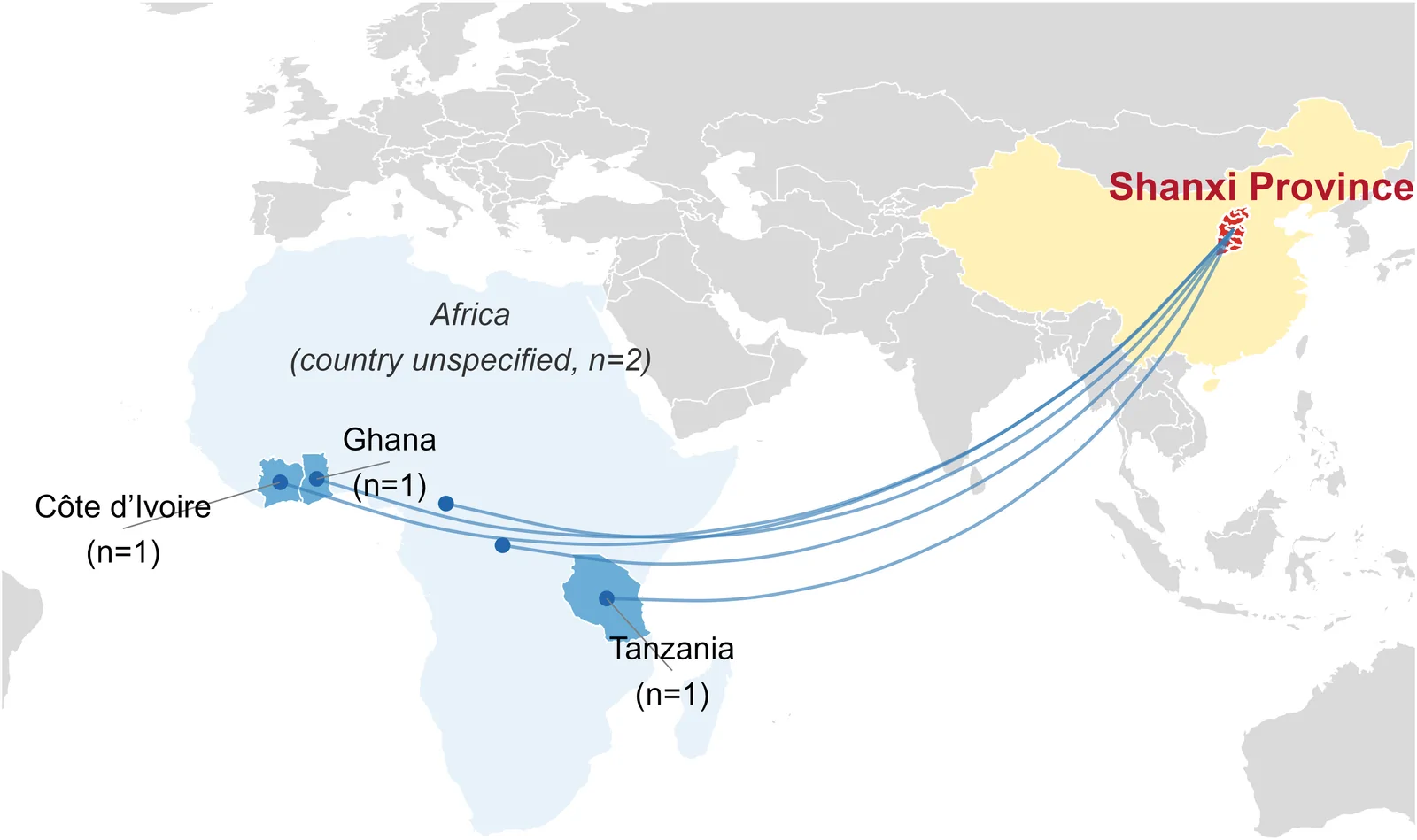
Geographic distribution of presumed malaria exposure among the five imported *Plasmodium falciparum* malaria. The map illustrates the presumed geographic exposure locations of the five patients and their relationship to Shanxi Province, China. One patient had returned from Côte d’Ivoire, one from Ghana, and one from Tanzania, whereas the country of exposure within Africa could not be further specified for two patients. Curved lines indicate the presumed routes from malaria-endemic regions in Africa to Shanxi Province.

Fever was the chief complaint in all five patients. The duration of fever before admission ranged from 5 to 14 days, and the maximum recorded body temperature ranged from 39.0 °C to 41.0 °C. Patient 3 had the longest febrile course before admission (14 days) and the highest recorded temperature (41.0 °C), whereas the remaining patients had fever durations of 5–7 days and maximum temperatures between 39.0 °C and 39.5 °C.

Accompanying manifestations varied among patients. Patient 1 reported headache, fatigue, profuse sweating, disturbance of consciousness, and cola-colored urine. Patient 2 presented mainly with nausea and poor appetite. Patient 3 experienced chills, shivering, myalgia, headache, and dark tea-colored urine. Patient 4 reported generalized weakness of the extremities, whereas Patient 5 presented with diarrhea, headache, vomiting, and bilateral lower-limb pain. Overall, although fever was the consistent presenting feature, the associated symptoms were heterogeneous, ranging from relatively nonspecific constitutional or gastrointestinal complaints to neurological manifestations and dark-colored urine. Detailed demographic, epidemiological, and presenting clinical characteristics are summarized in **Table 1**.

**Table 1:** Baseline demographic and clinical characteristics of five patients with imported malaria.

Variable	Patient 1	Patient 2	Patient 3	Patient 4	Patient 5
Patient ID	1	2	3	4	5
Gender	Male	Male	Male	Male	Male
Age (years)	51	28	42	30	26
Occupation	Farmer	Professional/technical worker	Professional/technical worker	Professional/technical worker	Professional/technical worker
Ethnicity	Han Chinese	Han Chinese	Han Chinese	Han Chinese	Han Chinese
Recent travel/residence history	Côte d’Ivoire	Africa	Tanzania	Ghana	Africa
Chief complaint	Fever	Fever	Fever	Fever	Fever
Duration of fever (days)	5	5	14	7	6
Maximum body temperature (°C)	39.4	39.5	41.0	39.0	39.5
Accompanying symptoms	Headache, fatigue, profuse sweating, disturbance of consciousness, and cola-colored urine	Nausea and poor appetite	Chills, shivering, myalgia, headache, and dark tea-colored urine	General weakness of the extremities	Diarrhea, headache, vomiting, and bilateral lower limb pain

### 3.2 Laboratory findings and major organ involvement

Initial laboratory findings demonstrated prominent hematological abnormalities and heterogeneous multiorgan involvement among the five patients (**Table 2**). Thrombocytopenia was present in all cases, with platelet counts ranging from 20 to 97 ×10^9^/L. Anemia was also common, particularly in Patients 3 and 4, whose hemoglobin levels were 71 and 80 g/L, respectively. White blood cell and neutrophil counts varied across the cohort and were not uniformly elevated.

**Table 2:** Initial laboratory findings of five patients with imported malaria at admission.

Parameter	Patient 1	Patient 2	Patient 3	Patient 4	Patient 5
Hematological parameters					
WBC (×10^9^/L)	7.6	5.6	4.4	4.7	9.6
N (×10^9^/L)	6.85	4.20	2.57	2.63	7.83
RBC (×10¹²/L)	3.46	3.86	2.21	2.47	3.61
HGB (g/L)	115	116	71	80	105
PLT (×10^9^/L)	23	44	97	33	20
Inflammatory markers					
IL-6 (pg/mL)	289.7	56.83	50.26	NR	11.24
PCT (ng/mL)	16.873	6.866	6.209	182.908	152.724
Liver injury and hemolysis-related parameters					
ALT (U/L)	17	63	30	16	84
AST (U/L)	51	59	46	39	155
ALP (U/L)	70	39	63	54	44
GGT (U/L)	34	35	36	29	117
TBIL (μmol/L)	40.4	35.4	34.6	21.2	139.8
DBIL (μmol/L)	10.6	12.7	13.1	11.3	82.7
IBIL (μmol/L)	29.8	22.7	21.5	9.9	57.1
LDH (U/L)	850	615	887	786	993
Renal function					
CRE (μmol/L)	62	80	65.9	664	214
Glucose metabolism					
GLU (mmol/L)	4.73	5.67	8.93	6.84	12.21
Coagulation parameters					
PT (s)	12.9	13.1	12.6	11.3	14.5
PTA (%)	82.4	75.9	83.9	103.1	66.3
INR	1.10	1.12	1.07	0.95	1.24
APTT (s)	30.6	26.0	31.4	27.1	39.4
D-D (mg/L FEU)	16.79	3.06	1.39	2585	129.03
FIB-C (g/L)	5.55	3.59	4.78	7.68	3.95

Inflammatory markers were markedly increased in several patients. Procalcitonin levels were elevated in all five cases, with particularly high values in Patients 4 and 5, whereas interleukin-6 was elevated in all patients with available measurements. Biochemical abnormalities mainly reflected variable hepatic and hemolysis-related involvement. Aspartate aminotransferase and lactate dehydrogenase were elevated in most patients, while bilirubin elevation was most pronounced in Patient 5, who had a total bilirubin concentration of 139.8 μmol/L. Patient 5 also showed the highest aminotransferase elevation, with an AST level of 155 U/L and ALT level of 84 U/L.

Renal dysfunction was a major feature in the more severe cases. Serum creatinine was markedly elevated in Patient 4 (664 μmol/L) and moderately elevated in Patient 5 (214 μmol/L), whereas renal function was largely preserved in the remaining patients. Coagulation abnormalities were variable. Patient 5 showed prolonged coagulation parameters, including an INR of 1.24 and APTT of 39.4 s, while D-dimer levels were substantially elevated in Patients 4 and 5.

Abdominal imaging findings also varied across the cohort. Patient 1 showed no significant abdominal abnormality. Fatty liver and splenomegaly were observed in Patient 2, while Patient 3 had fatty liver with minimal pelvic effusion. Patient 4 showed minimal pelvic effusion. Patient 5 had the most extensive abdominal abnormalities, including hepatomegaly with heterogeneous parenchymal changes, gallbladder inflammatory changes, focal common bile duct dilatation, and splenomegaly.

Overall, these findings demonstrated a spectrum of disease severity ranging from predominant hematological abnormalities to combined hepatic, renal, and coagulation involvement. Patients 4 and 5 showed the greatest degree of organ dysfunction, whereas the other cases were characterized mainly by thrombocytopenia, anemia, and biochemical evidence of systemic involvement.

### 3.3 mNGS findings and conventional parasitological assessment

mNGS was performed on peripheral blood samples obtained from all five patients during the clinical course. *P. falciparum* was identified in all cases (**Table 3**). Species-specific read counts varied markedly among patients, ranging from 121 in Patient 3 to 212,513 in Patient 1. The marked variation in sequencing reads did not correspond directly to clinical severity and was therefore not interpreted as a quantitative indicator of parasite burden. Despite this substantial variation in read counts, the relative abundance of *P. falciparum* remained consistently high across the five samples, ranging from 44.58% to 49.45%.

**Table 3:** mNGS findings in five patients with imported *Plasmodium falciparum* malaria.

Parameter	Patient 1	Patient 2	Patient 3	Patient 4	Patient 5
Sample type	Peripheral blood	Peripheral blood	Peripheral blood	Peripheral blood	Peripheral blood
Plasmodium species	*P. falciparum*	*P. falciparum*	*P. falciparum*	*P. falciparum*	*P. falciparum*
Species-specific reads	212,513	6,765	121	9,394	11,193
Relative abundance (%)	44.90	45.60	44.58	49.45	47.28
Genomic coverage (%)	49.63	3.94	0.0627	4.11	5.67
Mean sequencing depth	3.09	1.08	1.00	1.17	1.19
Other microorganisms detected	Human gammaherpesvirus 4	None	Human betaherpesvirus 6B	Human alphaherpesvirus 1	None
Depth ratio	0.61	0.94	1	0.88	0.87
Shannon index	1	1	1	1	1

Sequencing characteristics also differed considerably among patients. Genomic coverage ranged from 0.0627% to 49.63%, whereas mean sequencing depth ranged from 1.00 to 3.09. Patient 1 showed the highest read count and genomic coverage, whereas Patient 3 had the lowest sequencing signal, with 121 species-specific reads and a genomic coverage of 0.0627%. Nevertheless, *P. falciparum*-specific reads were detected across multiple regions of the reference genome in all five patients (**Figure 2**). Depth ratios ranged from 0.61 to 1.00, and the Shannon index was 1.00 in all cases, providing additional support for the genome-wide distribution and reliability of the detected *P. falciparum* sequences. Independent NCBI BLASTN verification of representative *P. falciparum*-assigned reads further supported the taxonomic assignment in all five patients. Representative alignment metrics, including query coverage, sequence identity, E-values, and corresponding NCBI accession numbers, are summarized (**Supplementary Table S1**).

**Figure 2:**
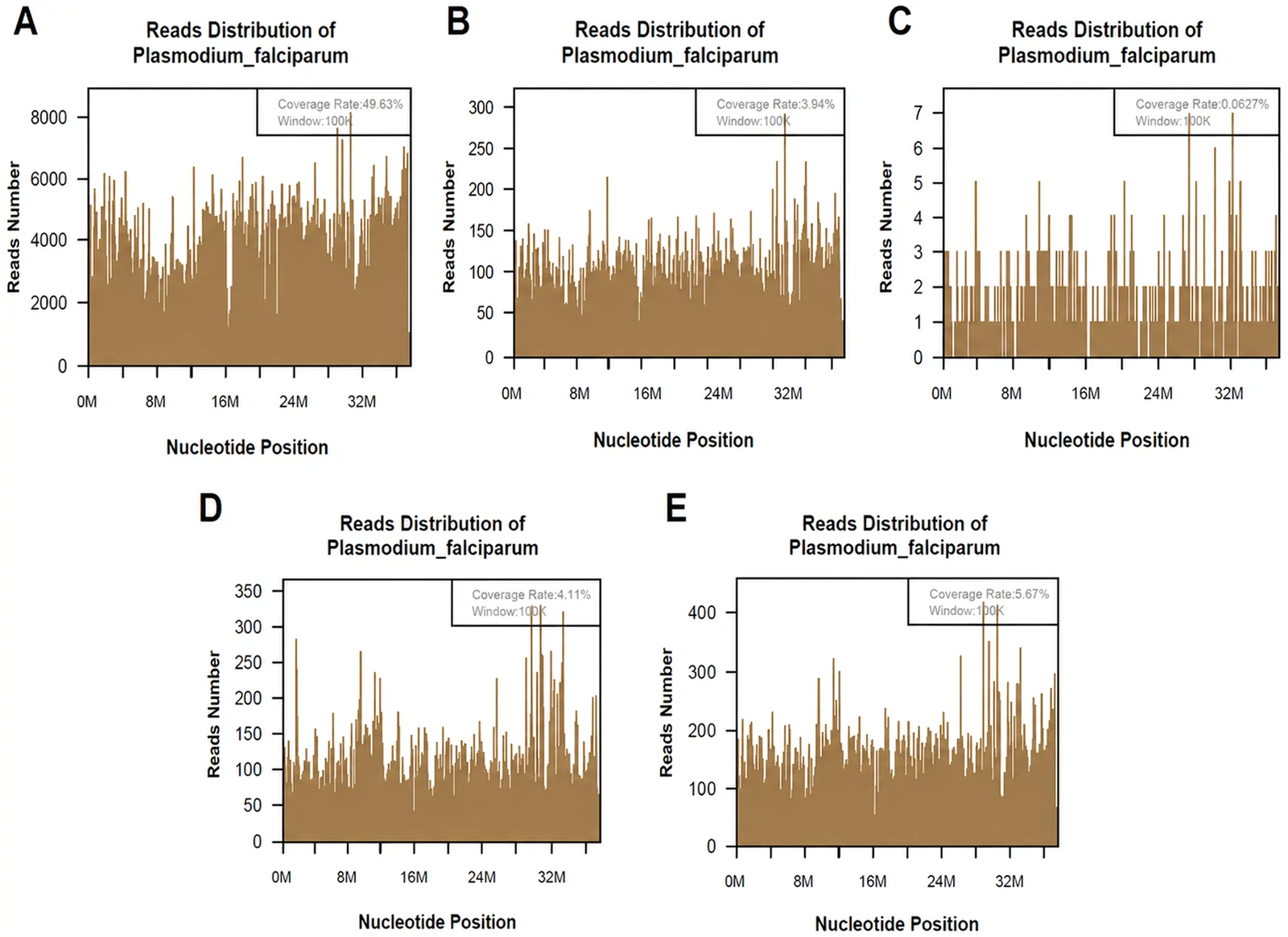
Genome-wide distribution of *Plasmodium falciparum*-specific sequencing reads in five patients. Panels **(A–E)** correspond to Patients 1–5, respectively. The horizontal axis represents nucleotide position across the reference genome, and the vertical axis represents the number of mapped reads within each genomic window. *P. falciparum*-specific reads were distributed across multiple genomic regions in all five patients, although the sequencing signal and genomic coverage varied substantially among individuals. The genomic coverage rates were 49.63%, 3.94%, 0.0627%, 4.11%, and 5.67% for Patients 1–5, respectively.

Additional microbial sequences were detected in three patients. Human gammaherpesvirus 4, human betaherpesvirus 6B, and human alphaherpesvirus 1 were detected in Patients 1, 3, and 4, respectively, whereas no additional microorganisms were reported in Patients 2 and 5. Based on the absence of corresponding clinical evidence and subsequent clinical assessment, these viral findings were not considered indicative of clinically relevant active coinfection.

Peripheral blood smear microscopy was also performed as part of the parasitological evaluation during hospitalization and demonstrated malaria parasites in the corresponding clinical context (**Figure 3**). Intraerythrocytic parasitic forms were visible to varying degrees across the five patients. Microscopy was used primarily for qualitative parasite detection and for documenting parasite persistence or clearance, whereas species-level identification of *P. falciparum* was supported primarily by mNGS together with epidemiological and clinical evidence. The detailed clinical contribution of mNGS at different stages of diagnosis and management is described in the following section.

**Figure 3:**
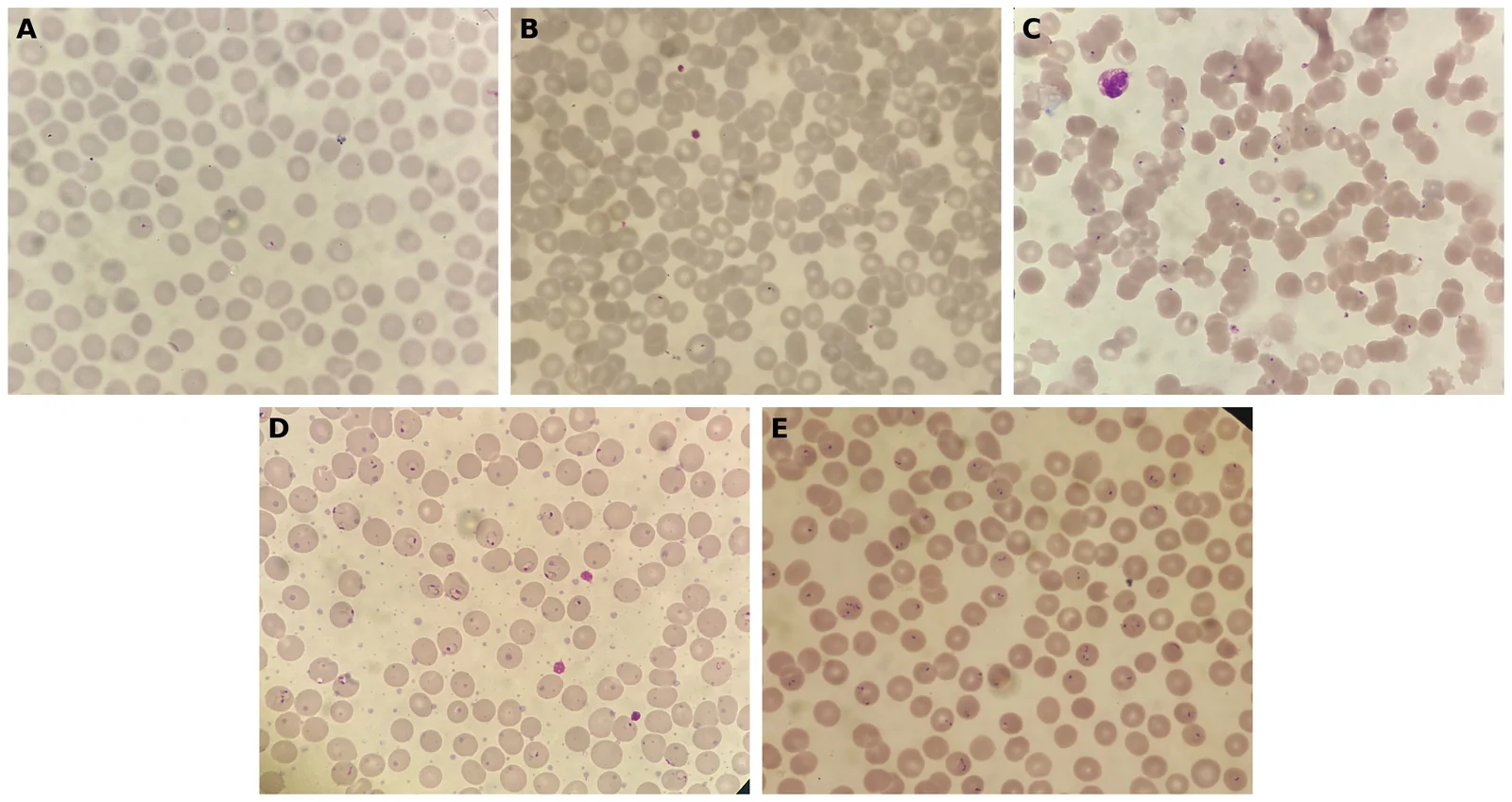
Representative peripheral blood smear images from five patients with imported malaria. Panels **(A–E)** correspond to Patients 1–5, respectively. Intraerythrocytic parasitic forms were visible to varying degrees across the five patients. These images are presented as qualitative microscopic evidence of malaria parasites and were not used independently for species-level identification.

### 3.4 Clinical contribution of mNGS in different diagnostic scenarios

The clinical contribution of mNGS varied according to the diagnostic context and disease course of individual patients (**Figure 4**). Rather than replacing conventional parasitological examinations, mNGS provided additional molecular evidence and assisted clinical decision-making in different stages of imported *P. falciparum* malaria management.

**Figure 4:**
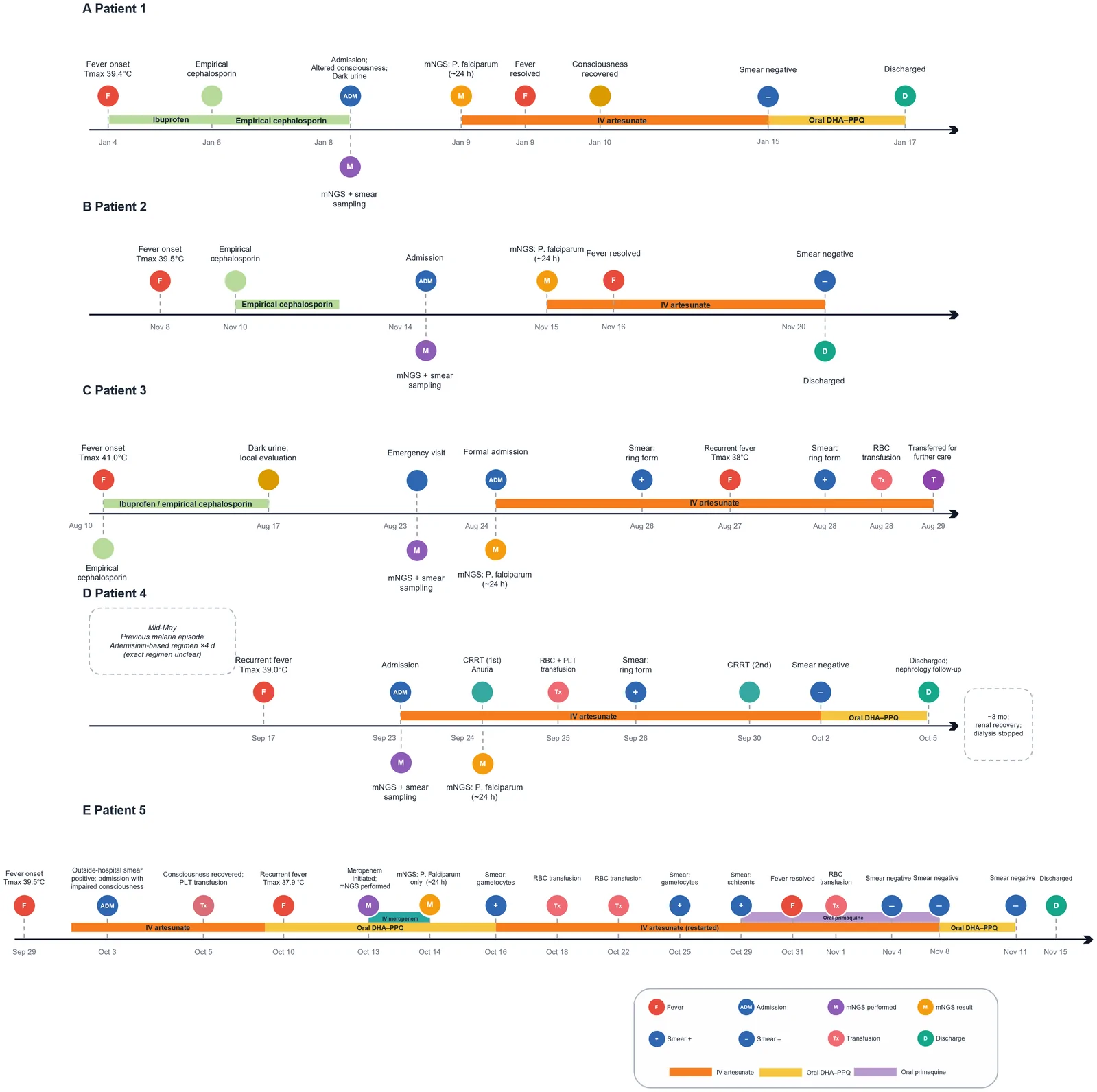
Clinical scenarios and timelines illustrating the role of mNGS in diagnosis and management of imported *Plasmodium falciparum* malaria. Panels **(A–E)** correspond to Patients 1–5, respectively. The timelines summarize the temporal relationships among fever onset, hospital admission, mNGS and peripheral blood smear sampling, availability of mNGS results, antimalarial treatment, blood transfusion or renal replacement therapy when required, parasite persistence or clearance on smear examination, and discharge or transfer. In Patients 1–4, mNGS was incorporated into the early etiological evaluation, whereas in Patient 5 it was performed during reassessment of recurrent fever after initial antimalarial treatment. ADM, admission; mNGS, metagenomic next-generation sequencing; IV, intravenous; DHA–PPQ, dihydroartemisinin–piperaquine; CRRT, continuous renal replacement therapy; RBC, red blood cell transfusion; PLT, platelet transfusion.

In Patients 1–4, mNGS was incorporated into the early etiological evaluation after admission because of the combination of epidemiological exposure, febrile illness, and laboratory abnormalities suggestive of malaria. *P. falciparum* sequences were identified in all four patients, providing molecular evidence supporting the diagnosis of imported falciparum malaria. The mNGS findings were interpreted together with peripheral blood smear examination and clinical manifestations, facilitating timely initiation or continuation of targeted antimalarial therapy.

Patient 5 represented a different diagnostic scenario in which mNGS was performed during clinical reassessment rather than initial diagnosis. Although malaria had already been identified by peripheral blood examination before admission, recurrent fever during the transition of antimalarial therapy raised concern for possible concomitant infection or alternative causes of persistent fever. mNGS detected *P. falciparum* without identifying additional clinically significant bacterial pathogens, providing additional microbiological information that did not support an alternative bacterial explanation at that time. Subsequent clinical evaluation incorporated the mNGS findings, repeated peripheral blood smear examinations, and treatment response to guide further management.

Additional microbial signals were detected in three patients during mNGS analysis, including human gammaherpesvirus 4, human betaherpesvirus 6B, and human alphaherpesvirus 1. However, these findings were not considered evidence of active viral infection based on the absence of compatible clinical manifestations and the overall disease course. These observations highlight that mNGS results require careful interpretation within the clinical context and should not be regarded as independent evidence of active infection.

Overall, mNGS provided diagnostic support across different clinical scenarios, including early pathogen identification and reassessment of atypical treatment courses. Its clinical value was greatest when integrated with epidemiological information, microscopy findings, and longitudinal clinical evaluation.

### 3.5 Treatment courses, dynamic changes, and clinical outcomes

All patients received antimalarial therapy according to current treatment recommendations. Intravenous artesunate was administered when parenteral therapy was required, followed by transition to oral DHA–PPQ after clinical stabilization. Additional supportive interventions, including red blood cell transfusion, platelet transfusion, and CRRT, were provided according to individual clinical conditions (**Figure 5**).

**Figure 5:**
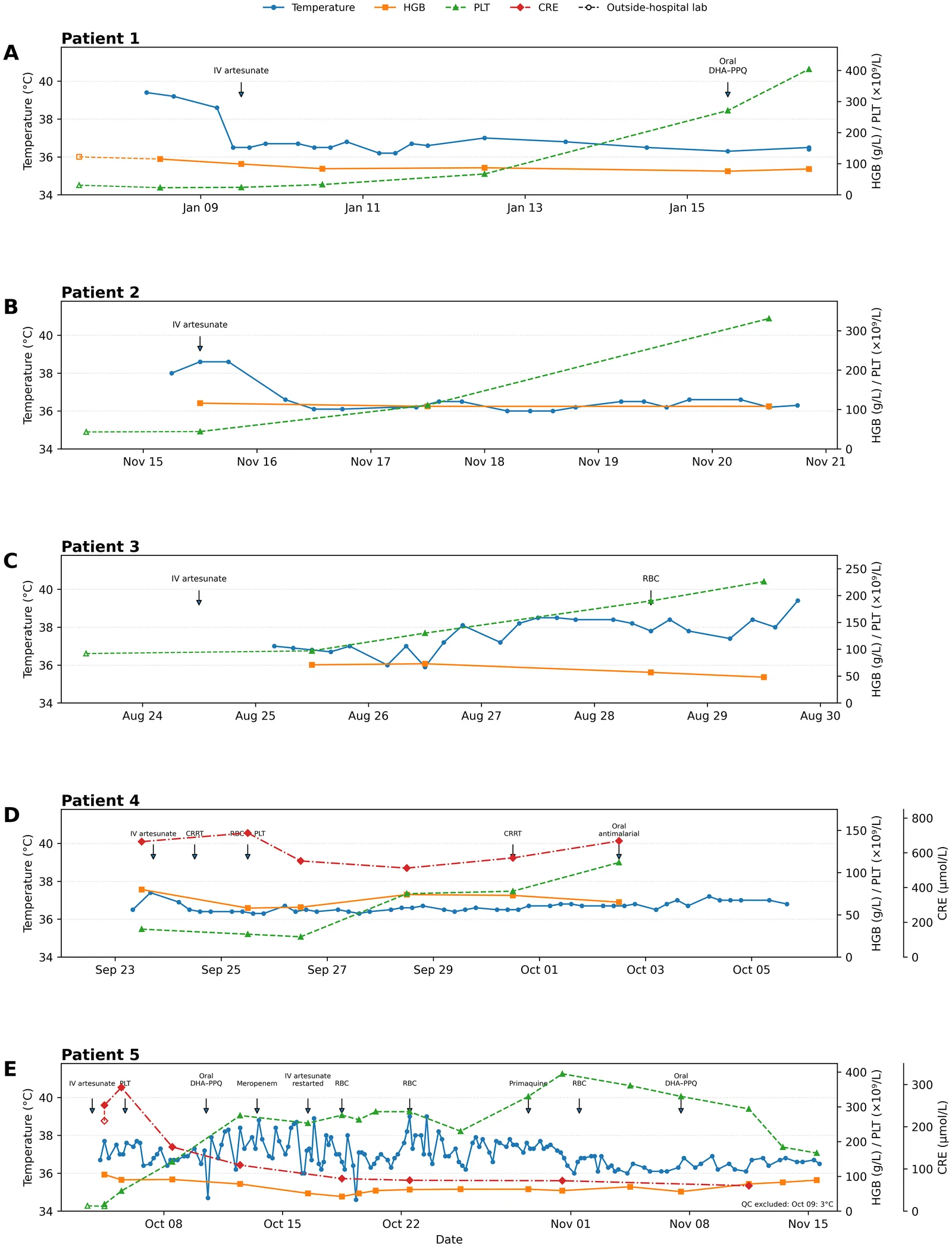
Longitudinal changes in body temperature and laboratory parameters during hospitalization in five patients with imported *Plasmodium falciparum* malaria. Panels **(A–E)** correspond to Patients 1–5, respectively. Serial changes in body temperature, hemoglobin concentration (HGB), platelet count (PLT), and serum creatinine (CRE) are shown according to the availability of clinical data. Major therapeutic interventions, including intravenous artesunate, oral dihydroartemisinin–piperaquine (DHA–PPQ), primaquine, red blood cell transfusion, platelet transfusion, and continuous renal replacement therapy, are indicated at the corresponding time points. Outside-hospital laboratory results are shown where available. The figure illustrates the heterogeneous and asynchronous recovery of fever, hematological abnormalities, and renal dysfunction among the five patients.

The clinical responses and laboratory trajectories varied according to disease severity. Patients 1 and 2 showed relatively rapid improvement after initiation of antimalarial therapy. In Patient 1, body temperature decreased shortly after intravenous artesunate administration, accompanied by gradual recovery of platelet counts. Neurological symptoms improved during hospitalization, and oral DHA–PPQ was introduced after clinical stabilization. Patient 2 achieved rapid defervescence with progressive platelet recovery and completed treatment without major complications.

More severe clinical courses were observed in Patients 3–5. Patient 3 experienced persistent fever during hospitalization with progressive anemia, requiring red blood cell transfusion. Although platelet counts gradually recovered, the patient was transferred to another hospital because of persistent clinical concerns, and subsequent outcome information was unavailable.

Patient 4 developed severe renal involvement, characterized by marked elevation of serum creatinine and oliguria, requiring repeated CRRT during hospitalization. Despite persistent renal dysfunction in the early phase, renal function gradually improved with continued antimalarial therapy and supportive management. Follow-up after discharge demonstrated progressive renal recovery, and dialysis was eventually discontinued.

Patient 5 had the most prolonged and complex clinical course. During hospitalization, the patient experienced recurrent fever, persistent smear positivity, progressive anemia, and repeated requirement for red blood cell transfusion. Dynamic monitoring showed gradual platelet recovery and improvement of renal function, whereas fever and parasite clearance required prolonged observation and treatment adjustment. After reassessment of the clinical course, intravenous artesunate was restarted and primaquine was added. Subsequently, fever resolved, peripheral blood smear examinations became negative, and the patient was transitioned to oral therapy before discharge.

Longitudinal monitoring demonstrated that clinical recovery was not synchronous among different disease indicators. Fever resolution, parasite clearance, hematological recovery, and renal function improvement followed different trajectories, particularly in patients with severe malaria manifestations. All patients survived to discharge or transfer, and no in-hospital deaths occurred.

## 4 Discussion

This case series describes five imported *P. falciparum* malaria cases managed in a malaria-free region of northern China and illustrates the heterogeneity of both clinical presentation and diagnostic pathways. All patients had recent exposure to malaria-endemic regions in Africa, but their clinical courses ranged from relatively uncomplicated febrile illness to neurological involvement, severe hematological abnormalities, AKI, and prolonged parasite positivity on peripheral blood smear. mNGS identified *P. falciparum* in all five patients; however, its clinical relevance was not primarily related to replacing conventional malaria diagnosis, but rather to providing additional microbiological evidence in situations where diagnostic uncertainty persisted or disease evolution was atypical. In Patients 1–4, mNGS formed part of the early etiological work-up together with conventional parasitological examination, whereas in Patient 5 it was used later for etiological reassessment when recurrent fever raised concern for a concomitant infection. These observations highlight two practical issues in post-elimination settings: imported malaria must remain in the differential diagnosis of febrile returning travelers, and mNGS should be integrated with, rather than viewed as a substitute for, established parasitological methods. The diagnostic challenges illustrated by these cases may also be relevant to other malaria-free regions where local transmission has been interrupted and clinical exposure to malaria has decreased. In such settings, maintaining clinical awareness and diagnostic proficiency for imported malaria represents an ongoing challenge after elimination rather than a limitation of individual healthcare systems (Mischlinger et al., 2020).

Imported malaria remains particularly challenging in areas where indigenous transmission has been eliminated. As malaria becomes uncommon in routine clinical practice, both clinicians and laboratory personnel may have less opportunity to recognize the disease promptly, increasing the risk of diagnostic delay in patients whose travel history is incomplete or initially overlooked (Bastaki et al., 2018; Weiland, 2023). The five patients in our series had experienced fever for 5–14 days before admission, despite all having epidemiological exposure compatible with imported malaria. Early manifestations of falciparum malaria are often nonspecific and may include fever, chills, headache, fatigue, gastrointestinal symptoms, or other systemic complaints that overlap with common bacterial or viral infections (Ashley et al., 2018). Severe disease may subsequently involve the central nervous system, hematological system, kidneys, and coagulation pathways (Bittaye et al., 2022; White, 2022). Delays in imported-malaria diagnosis may therefore reflect both patient-related and healthcare-provider factors, including delayed care seeking, incomplete recognition of travel-related risk, and limited experience with malaria in non-endemic settings (Bittaye et al., 2022; Lu et al., 2022). These observations reinforce the continued importance of obtaining a detailed travel history in patients with unexplained fever after international travel. Beyond travel history, another important issue in non-endemic settings is the gradual loss of malaria-specific diagnostic experience. Because imported cases are sporadic, clinicians may have limited opportunities to recognize malaria presentations, while laboratories may face difficulties maintaining expertise in parasite identification and parasitemia assessment (Calderaro et al., 2021).

These constraints had specific implications for parasitological assessment in our clinical setting. Peripheral blood smear examination was used primarily for qualitative parasite detection, whereas standardized quantitative parasitemia measurement was not routinely available. Serial smears were performed according to clinical need rather than according to a predefined daily monitoring schedule; consequently, complete daily parasite-density data and parasite-clearance kinetics could not be reconstructed retrospectively. This limitation is clinically relevant because quantitative parasitemia provides important information for assessing disease severity and treatment response and may contribute to decisions regarding continuation of parenteral therapy and transition to oral treatment. In our patients, such treatment decisions therefore relied primarily on clinical improvement, qualitative smear findings, and changes in routine laboratory parameters rather than on standardized parasite-density thresholds. In addition, although malaria parasites could be recognized microscopically, reliable species-level identification based solely on erythrocyte morphology was not consistently feasible in routine practice. Thus, microscopy in this series was mainly used to document parasite presence, persistence, and clearance, while species-level identification of *P. falciparum* was supported primarily by mNGS together with epidemiological and clinical evidence.

The clinical findings in our patients were consistent with the broad systemic involvement reported in falciparum malaria, but disease severity varied considerably. Thrombocytopenia was present in all five patients, while anemia was particularly prominent in Patients 3 and 4. Neurological involvement and dark-colored urine occurred in Patient 1, and Patients 4 and 5 developed marked renal dysfunction. Such heterogeneity is clinically relevant because the severity of malaria cannot be inferred from fever alone. Microscopy remains fundamental for malaria diagnosis because it provides direct visualization of parasites and permits subsequent assessment of parasite persistence or clearance; however, its performance depends on parasite density, sampling and examiner expertise, which may be more challenging to maintain in non-endemic laboratories (Calderaro et al., 2021; Williams and Drennan, 2022). The practical diagnostic problem in malaria-free regions is therefore not simply whether an effective conventional method exists, but whether malaria is considered sufficiently early and whether appropriate parasitological expertise is readily available when a patient first presents.

Within this context, the value of mNGS in our series should be interpreted as complementary rather than substitutive. mNGS enables broad-range detection without requiring a predefined pathogen-specific target and has been increasingly applied to diagnostically challenging, atypical, or unexpected infectious diseases (Simner et al., 2018; Chiu and Miller, 2019; Wilson et al., 2019; Rong et al., 2026). In Patients 1–4, sequencing was incorporated into early etiological investigation and provided molecular identification of *P. falciparum* alongside blood smear examination. The sequencing characteristics varied markedly: species-specific reads ranged from 121 to 212,513, and genomic coverage ranged from 0.0627% to 49.63%. Importantly, these quantitative differences should not be interpreted in isolation. Patient 3, for example, had the lowest read count and genomic coverage, yet the detected sequences showed a distribution compatible with *P. falciparum* and were supported by the epidemiological and parasitological findings. This is consistent with the broader principle that mNGS read counts alone do not necessarily establish or exclude clinically relevant infection and should be interpreted together with the clinical context (Miao et al., 2018; Gu et al., 2019). In this context, mNGS may serve as an additional objective microbiological approach when clinical suspicion remains high but diagnostic pathways are complicated by atypical presentations or limited local expertise. However, its findings should always be integrated with conventional parasitological examination rather than interpreted independently (Gu et al., 2019).

Patient 5 illustrates a different and potentially more informative application of mNGS. In this patient, malaria had already been recognized and antimalarial therapy had been initiated before sequencing was performed. Recurrent fever and headache during transition to oral therapy raised concern for a concomitant bacterial infection, resulting in empirical antibacterial treatment and further etiological investigation. mNGS again detected *P. falciparum* but did not identify an additional clinically relevant bacterial pathogen. This result did not by itself exclude bacterial coinfection, but it provided additional microbiological evidence that did not support an alternative bacterial explanation. Subsequent microscopy continued to demonstrate parasite forms, and the antimalarial regimen was therefore reassessed according to the combined parasitological and clinical course. This case also illustrates an important limitation of broad-range sequencing: detection of microbial nucleic acid is not synonymous with active infection (Miao et al., 2018; Gu et al., 2019). Human gammaherpesvirus 4, human betaherpesvirus 6B, and human alphaherpesvirus 1 sequences were detected in three other patients, but there was no corresponding clinical evidence supporting active viral disease. Herpesvirus-family sequences may represent latent infection, reactivation, or incidental detection, particularly when sequencing is applied without a predefined target (Rong et al., 2026). Accordingly, indiscriminate interpretation of every detected organism may increase diagnostic uncertainty and potentially lead to unnecessary antimicrobial treatment (Chiu and Miller, 2019). In our study, mNGS findings were therefore interpreted jointly with epidemiological exposure, clinical manifestations, microscopy, laboratory abnormalities, and treatment response. Patient 5 also highlights the difficulty of evaluating treatment response in imported falciparum malaria. Recurrent fever and persistent parasite detection after initial therapy may result from delayed parasite clearance, recrudescence, inadequate drug exposure, treatment-related factors, or possible reduced susceptibility; however, distinguishing these possibilities requires systematic parasitological monitoring and, when available, molecular resistance assessment (Lalloo et al., 2016). In malaria-free regions, where quantitative parasitemia monitoring and resistance surveillance may not be routinely accessible, clinicians may need to integrate multiple sources of evidence when making treatment decisions.

The severe cases in our series further emphasize the importance of monitoring organ dysfunction throughout the course of falciparum malaria. Severe malaria can involve neurological impairment, anemia, thrombocytopenia, AKI, metabolic disturbances, and coagulation abnormalities (Ashley et al., 2018; White, 2022). Non-immune travelers from non-endemic settings may be particularly susceptible to severe disease because they lack the partial protection associated with repeated exposure in endemic populations (Lalloo et al., 2016). Renal involvement was most pronounced in Patient 4, who developed anuria and marked creatinine elevation requiring repeated CRRT. Malaria-associated AKI is multifactorial and has been linked to hemolysis, microvascular and endothelial injury, inflammatory responses, and parasite-related circulatory disturbances (Hegde et al., 2024). Although severe AKI is associated with adverse outcomes, substantial renal recovery may occur with effective antimalarial therapy and appropriate organ support (Akafity et al., 2024; Hegde et al., 2024). This was observed in Patient 4, whose renal function continued to improve after discharge until dialysis was eventually discontinued. Hematological recovery also differed between patients. Thrombocytopenia improved progressively in most cases, whereas anemia was more prolonged and required red blood cell transfusion in selected patients. Both parasite-mediated erythrocyte destruction and non-parasitized erythrocyte loss may contribute to malaria-associated anemia (White, 2018).

Treatment in this series was performed according to recommended malaria-management principles, with intravenous artesunate used for parenteral treatment and oral therapy introduced when clinically appropriate. Intravenous artesunate remains central to the management of severe falciparum malaria because of its rapid parasiticidal activity and established role in contemporary treatment strategies (Salkeld et al., 2024). Nevertheless, our longitudinal observations show that fever resolution, parasite clearance, hematological recovery, and organ recovery do not necessarily occur simultaneously. Patient 5 had recurrent fever and persistent parasite forms despite initial clinical improvement, while Patient 4 experienced prolonged renal dysfunction despite control of fever and recovery of other parameters. Thus, treatment response in imported falciparum malaria should be evaluated using a combination of clinical status, serial parasitological assessment, and organ-function monitoring rather than a single marker of improvement.

This study has several strengths. It provides longitudinal real-world data on imported falciparum malaria from a malaria-free region and captures different clinical contexts in which mNGS was used, including early etiological investigation and reassessment during an atypical treatment course. The integration of epidemiological history, sequencing characteristics, parasitological findings, treatment timelines, and longitudinal hematological and renal changes allowed the disease course to be examined beyond a single diagnostic time point.

Several limitations should nevertheless be acknowledged. This was a retrospective single-center case series involving only five patients, and the findings should therefore be considered descriptive rather than evidence that mNGS is superior to conventional malaria diagnostics. The absence of a comparator group prevented formal assessment of the incremental diagnostic benefit of mNGS, and additional microbial signals were not systematically validated by independent molecular assays. Furthermore, follow-up information was unavailable after transfer in Patient 3. mNGS detects parasite-derived nucleic acids and cannot reliably distinguish viable *P. falciparum* from nonviable or residual parasite material after treatment. Therefore, a positive mNGS result should not be interpreted as direct evidence of active parasitemia or viable parasite burden, and serial microscopy remains necessary for assessing parasite persistence and clearance during treatment. In addition, quantitative parasitemia was not routinely measured and peripheral blood smears were not obtained according to a predefined daily schedule; therefore, daily parasite-density trajectories and parasite-clearance kinetics could not be reconstructed retrospectively. Larger multicenter studies will be required to clarify the specific clinical settings in which mNGS provides the greatest additional value for the diagnosis and management of imported malaria.

## 5 Conclusion

Imported *P. falciparum* malaria remains clinically challenging in malaria-free regions because recognition may be delayed and disease severity can vary substantially. In this five-case series, mNGS complemented conventional parasitological assessment by contributing to early etiological identification in some patients and to diagnostic reassessment in a patient with recurrent fever during treatment. Its findings were most useful when interpreted together with travel history, microscopy, clinical evolution, and treatment response rather than as standalone evidence. These cases support a role for mNGS as an adjunctive tool in the diagnosis and management of imported falciparum malaria, particularly when the presentation or subsequent clinical course is atypical. These cases may also serve as a clinical reference for other malaria-free regions where imported malaria remains uncommon but potentially life-threatening.

## Data Availability

The data analyzed in this study is subject to the following licenses/restrictions: The raw data supporting the conclusions of this article will be made available by the authors, without undue reservation. Requests to access these datasets should be directed to Bin Niu, sxtynb1997@163.com.

## References

[B1] Akafity G. Kumi N. Ashong J. (2024). Diagnosis and management of malaria in the intensive care unit. J. Intensive Med. 4, 3–15. doi: 10.1016/j.jointm.2023.09.002 38263976 PMC10800773

[B2] Ashley E. A. Pyae Phyo A. Woodrow C. J. (2018). Malaria. Lancet 391, 1608–1621. doi: 10.1016/S0140-6736(18)30324-6 29631781

[B3] Bastaki H. Carter J. Marston L. Cassell J. Rait G. (2018). Time delays in the diagnosis and treatment of malaria in non-endemic countries: A systematic review. Travel Med. Infect. Dis. 21, 21–27. doi: 10.1016/j.tmaid.2017.12.002 29225124

[B4] Bittaye S. O. Jagne A. Jaiteh L. E. Nadjm B. Amambua-Ngwa A. Sesay A. K. . (2022). Clinical manifestations and outcomes of severe malaria in adult patients admitted to a tertiary hospital in the Gambia. Malar. J. 21, 270. doi: 10.1186/s12936-022-04294-4 36131306 PMC9491657

[B5] Calderaro A. Montecchini S. Buttrini M. Piccolo G. Rossi S. Arcangeletti M. C. . (2021). Malaria diagnosis in non-endemic settings: The European experience in the last 22 years. Microorganisms 9, 2265. doi: 10.3390/microorganisms9112265 34835391 PMC8620059

[B6] Chiu C. Y. Miller S. A. (2019). Clinical metagenomics. Nat. Rev. Genet. 20, 341–355. doi: 10.1038/s41576-019-0113-7 30918369 PMC6858796

[B7] Edward P. Handel A. S. (2021). Metagenomic next-generation sequencing for infectious disease diagnosis: A review of the literature with a focus on pediatrics. J. Pediatr. Infect. Dis. Soc 10, S71–S77. doi: 10.1093/jpids/piab104 34951466

[B8] Gu W. Miller S. Chiu C. Y. (2019). Clinical metagenomic next-generation sequencing for pathogen detection. Annu. Rev. Pathol. 14, 319–338. doi: 10.1146/annurev-pathmechdis-012418-012751 30355154 PMC6345613

[B9] Hegde A. Chhallani A. K. Gupta B. Kadapatti K. Karnad D. Maheshwarappa H. M. . (2024). ISCCM position statement on the management of severe malaria in intensive care unit. Indian J. Crit. Care Med. 28, S59–S66. doi: 10.5005/jp-journals-10071-24765 39234225 PMC11369926

[B10] Lalloo D. G. Shingadia D. Bell D. J. Beeching N. J. Whitty C. J. M. Chiodini P. L. . (2016). UK malaria treatment guidelines 2016. J. Infect. 72, 635–649. doi: 10.1016/j.jinf.2016.02.001 26880088 PMC7132403

[B11] Li W. Zhang X. Feng J. Zhang T. Xu X. Jiang J. . (2021). Evaluation of the combination of rapid diagnostic tests and microscopy for imported malaria surveillance in Anhui Province, China. Acta Trop. 222, 106042. doi: 10.1016/j.actatropica.2021.106042 34252385

[B12] Liu B. Zhang T. Wang D. Xia S. Li W. Zhang X. . (2024). Profile and determinants for complications of imported malaria in 5 Chinese provinces from 2014 to 2021: Retrospective analysis. JMIR Public Health Surveill. 10, e52089. doi: 10.2196/52089 39212596 PMC11378694

[B13] Liu Y. Ma Y. (2024). Clinical applications of metagenomics next-generation sequencing in infectious diseases. J. Zhejiang Univ.-Sci. B 25, 471–484. doi: 10.1631/jzus.B2300029 38910493 PMC11199093

[B14] Lu G. Cao Y. Chai L. Li Y. Li S. Heuschen A. K. . (2022). Barriers to seeking health care among returning travellers with malaria: A systematic review. Trop. Med. Int. Health 27, 28–37. doi: 10.1111/tmi.13698 34748264

[B15] Miao Q. Ma Y. Wang Q. Pan J. Zhang Y. Jin W. . (2018). Microbiological diagnostic performance of metagenomic next-generation sequencing when applied to clinical practice. Clin. Infect. Dis. 67, S231–S240. doi: 10.1093/cid/ciy693 30423048

[B16] Mischlinger J. Rönnberg C. Álvarez-Martínez M. J. Bühler S. Paul M. Schlagenhauf P. . (2020). Imported malaria in countries where malaria is not endemic: A comparison of semi-immune and nonimmune travelers. Clin. Microbiol. Rev. 33, e00104-19. doi: 10.1128/CMR.00104-19 32161068 PMC7067581

[B17] National Center for Infectious Diseases Writing Group (2022). Guidelines for the diagnosis and treatment of malaria. Chin. J. Parasitol. Parasitic Dis. 40, 419. doi: 10.12140/j.issn.1000-7423.2022.04.001

[B18] Rong R. Long Y. Li Y. Lin L. Yang J. Hu Z. . (2026). Metagenomic and targeted next-generation sequencing in infectious disease diagnostics: Current applications, challenges, and future perspectives. Diagnostics 16, 991. doi: 10.3390/diagnostics16070991 41975703 PMC13073026

[B19] Salkeld J. Duncan A. Minassian A. M. (2024). Malaria: Past, present and future. Clin. Med. 24, 100258. doi: 10.1016/j.clinme.2024.100258 39490741 PMC11605449

[B20] Simner P. J. Miller S. Carroll K. C. (2018). Understanding the promises and hurdles of metagenomic next-generation sequencing as a diagnostic tool for infectious diseases. Clin. Infect. Dis. 66, 778–788. doi: 10.1093/cid/cix881 29040428 PMC7108102

[B21] Weiland A. S. (2023). Recent advances in imported malaria pathogenesis, diagnosis, and management. Curr. Emerg. Hosp. Med. Rep. 11, 49–57. doi: 10.1007/s40138-023-00264-5 37213266 PMC10091340

[B22] White N. J. (2018). Anaemia and malaria. Malar. J. 17, 371. doi: 10.1186/s12936-018-2509-9 30340592 PMC6194647

[B23] White N. J. (2022). Severe malaria. Malar. J. 21, 284. doi: 10.1186/s12936-022-04301-8 36203155 PMC9536054

[B24] Williams L. Drennan V. M. (2022). Evaluating the efficacy of rapid diagnostic tests for imported malaria in high income countries: A systematic review. Int. Emerg. Nurs. 60, 101110. doi: 10.1016/j.ienj.2021.101110 34953438

[B25] Wilson M. R. Sample H. A. Zorn K. C. Arevalo S. Yu G. Neuhaus J. . (2019). Clinical metagenomic sequencing for diagnosis of meningitis and encephalitis. N. Engl. J. Med. 380, 2327–2340. doi: 10.1056/NEJMoa1803396 31189036 PMC6764751

[B26] Zhu Y. Restrepo A. C. Wang H. B. Mills D. J. Liang R. R. Liu Z. B. . (2024). Malaria cases in China acquired through international travel, 2013-2022. J. Travel Med. 31, taae056. doi: 10.1093/jtm/taae056 38591791 PMC11646087

