# Supplementary material for: Clinical contribution of mNGS in the diagnosis and management of imported falciparum malaria in a malaria-free region: a five-case series from northern China
Source: Front Cell Infect Microbiol. 2026 Sep 24;16:1978670. doi: 10.3389/fcimb.2026.1978670 (PMC13645569; doi:10.3389/fcimb.2026.1978670)
Supplement: Supplementary file 1 [file Table1.docx]

**Supplementary Table S1 NCBI BLASTN verification of representative mNGS reads assigned to *Plasmodium falciparum***

| **Patient** | **Read ID** | **Query length (bp)** | **Description** | **Scientific name** | **Max Score** | **Query cover (%)** | **E-value** | **Identity (%)** | **NCBI accession number** |
| --- | --- | --- | --- | --- | --- | --- | --- | --- | --- |
| Patient 1 | P1-R1 | 43bp | Plasmodium falciparum 3D7 EMP1-trafficking protein (PF3D7_0731100), partial mRNA | [*Plasmodium falciparum*](https://www.ncbi.nlm.nih.gov/Taxonomy/Browser/wwwtax.cgi?command=show&mode=node&id=5833&lvl=) | 80.5 | 100% | 7e-12 | 100.00% | XM_001349168.1 |
| Patient 1 | P1-R2 | 43bp | Plasmodium falciparum 3D7 LCCL domain-containing protein (PF3D7_1451600), partial mRNA | *Plasmodium falciparum* | 80.5 | 100% | 7e-12 | 100.00% | XM_001348629.2 |
| Patient 1 | P1-R3 | 43bp | Plasmodium falciparum isolate 9A_03 glutamate dehydrogenase gene, partial cds | [*Plasmodium falciparum*](https://www.ncbi.nlm.nih.gov/Taxonomy/Browser/wwwtax.cgi?command=show&mode=node&id=5833&lvl=) | 80.5 | 100% | 7e-12 | 100.00% | MK920432.1 |
| Patient 1 | P1-R4 | 43bp | Plasmodium falciparum isolate Pf114_36 RIFIN (rif) pseudogene, partial sequence | *Plasmodium falciparum* | 80.5 | 100% | 7e-12 | 100.00% | [DQ265267.1](https://www.ncbi.nlm.nih.gov/nucleotide/DQ265267.1?report=genbank&log$=nucltop&blast_rank=1&RID=A6JYAG3Y016) |
| Patient 1 | P1-R5 | 43bp | P.falciparum alpha-tubulin II gene, complete cds | *Plasmodium falciparum* | 80.5 | 100% | 7e-12 | 100.00% | M34390.1 |
| Patient 2 | P2-R1 | 41bp | Plasmodium falciparum repetitive sequence DNA | *Plasmodium falciparum* | 76.8 | 100% | 7e-11 | 100.00% | M93045.1 |
| Patient 2 | P2-R2 | 41bp | Plasmodium falciparum isolate 109-19 erythrocyte membrane protein 1 (EMP1) mRNA, partial cds | *Plasmodium falciparum* | 76.8 | 100% | 7e-11 | 100.00% | MW014643.1 |
| Patient 2 | P2-R3 | 41bp | Plasmodium falciparum isolate 1974-2 erythrocyte membrane protein 1 (var) gene, exon 1 and partial cds | [*Plasmodium falciparum*](https://www.ncbi.nlm.nih.gov/Taxonomy/Browser/wwwtax.cgi?command=show&mode=node&id=5833&lvl=) | 76.8 | 100% | 7e-11 | 100.00% | [KX154900.1](https://www.ncbi.nlm.nih.gov/nucleotide/KX154900.1?report=genbank&log$=nucltop&blast_rank=1&RID=A60RT2U6016) |
| Patient 2 | P2-R4 | 41bp | Plasmodium falciparum isolate Pf134_02 RIFIN (rif) gene, exon 2 and partial cds | *Plasmodium falciparum* | 76.8 | 100% | 7e-11 | 100.00% | DQ265313.1 |
| Patient 2 | P2-R5 | 41bp | Plasmodium falciparum variant-specific surface protein (varph17) gene, partial cds | *Plasmodium falciparum* | 71.3 | 100% | 3e-9 | 97.56% | AF050740.1 |
| Patient 3 | P3-R1 | 43bp | Plasmodium falciparum isolate 20_Seq7-B varB gene, partial sequence | *Plasmodium falciparum* | 75.0 | 100% | 3e-10 | 97.67% | [JF759186.1](https://www.ncbi.nlm.nih.gov/nucleotide/JF759186.1?report=genbank&log$=nucltop&blast_rank=1&RID=A6605ABG014) |
| Patient 3 | P3-R2 | 43bp | Plasmodium falciparum 3D7 gametocyte-specific protein (PF3D7_1038400), partial mRNA | *Plasmodium falciparum* | 80.5 | 100% | 7e-12 | 100.00% | XM_001347622.2 |
| Patient 3 | P3-R3 | 43bp | Plasmodium falciparum isolate TR136 erythrocytic binding antigen gene, partial cds | *Plasmodium falciparum* | 80.5 | 100% | 7e-12 | 100.00% | MT507498.1 |
| Patient 3 | P3-R4 | 43bp | Plasmodium falciparum isolate 132Indo mitochondrion, complete genome | *Plasmodium falciparum* | 80.5 | 100% | 7e-12 | 100.00% | KY923486.1 |
| Patient 3 | P3-R5 | 43bp | Plasmodium falciparum 3D7 GBPH protein (PF3D7_1401000), partial mRNA | *Plasmodium falciparum* | 80.5 | 100% | 7e-12 | 100.00% | XM_001348149.1 |
| Patient 4 | P4-R1 | 43bp | Plasmodium falciparum 3D7 stevor (PF3D7_0402600), partial mRNA | *Plasmodium falciparum* | 80.5 | 100% | 7e-12 | 100.00% | [XM_001351303.1](https://www.ncbi.nlm.nih.gov/nucleotide/XM_001351303.1?report=genbank&log$=nucltop&blast_rank=1&RID=A6BCNGM6016) |
| Patient 4 | P4-R2 | 43bp | [Plasmodium falciparum isolate 132Indo mitochondrion, complete genome](https://blast.ncbi.nlm.nih.gov/Blast.cgi#alnHdr_1369113312) | *Plasmodium falciparum* | 80.5 | 100% | 7e-12 | 100.00% | KY923486.1 |
| Patient 4 | P4-R3 | 43bp | Plasmodium falciparum Pfa_SERA6 gene for putative papain-like cysteine prorease, complete cds, strain: 7G8 | *Plasmodium falciparum* | 80.5 | 100% | 7e-12 | 100.00% | [AB733785.1](https://www.ncbi.nlm.nih.gov/nucleotide/AB733785.1?report=genbank&log$=nucltop&blast_rank=1&RID=A6BCNGM6016) |
| Patient 4 | P4-R4 | 43bp | Plasmodium falciparum isolate NPD_19510016 lncRNA, complete sequence | *Plasmodium falciparum* | 80.5 | 100% | 7e-12 | 100.00% | PZ538039.1 |
| Patient 4 | P4-R5 | 43bp | Plasmodium falciparum 3D7 erythrocyte membrane protein 1, PfEMP1 (PF3D7_0700100), partial mRNA | *Plasmodium falciparum* | 80.5 | 100% | 7e-12 | 100.00% | XM_002808860.1 |
| Patient 5 | P5-R1 | 43bp | Plasmodium falciparum 3D7 rifin (PF3D7_0300800), partial mRNA | *Plasmodium falciparum* | 80.5 | 100% | 7e-12 | 100.00% | [XM_001351049.1](https://www.ncbi.nlm.nih.gov/nucleotide/XM_001351049.1?report=genbank&log$=nucltop&blast_rank=1&RID=A6D4DY2C016) |
| Patient 5 | P5-R2 | 43bp | Plasmodium falciparum isolate 132Indo mitochondrion, complete genome | *Plasmodium falciparum* | 80.5 | 100% | 7e-12 | 100.00% | KY923486.1 |
| Patient 5 | P5-R3 | 43bp | Plasmodium falciparum isolate 6_Seq5-C varC gene, partial sequence | *Plasmodium falciparum* | 80.5 | 100% | 7e-12 | 100.00% | [JF759229.1](https://www.ncbi.nlm.nih.gov/nucleotide/JF759229.1?report=genbank&log$=nucltop&blast_rank=1&RID=A6EG2XBP016) |
| Patient 5 | P5-R4 | 43bp | Plasmodium falciparum isolate Pf42_35 RIFIN (rif) gene, exon 2 and partial cds | *Plasmodium falciparum* | 75.0 | 93% | 3e-10 | 100.00% | DQ265162.1 |
| Patient 5 | P5-R5 | 43bp | Plasmodium falciparum (strain FCR3) variant-specific surface protein (var-2, var-3) genes, complete cds's | *Plasmodium falciparum* | 73.1 | 91% | 1e-09 | 100.00% | L40609.1 |
